# Immune checkpoint inhibitors and the pediatric rheumatologist: a pediatric needs assessment

**DOI:** 10.1186/s12969-025-01127-x

**Published:** 2025-07-15

**Authors:** John Storwick, Carrie Ye, Shahin Jamal, Nancy Maltez, Mercedes Chan

**Affiliations:** 1 Division of Pediatric Rheumatology, British Columbia Children’s Hospital, The University of British Columbia, Vancouver, BC Canada; 2 Department of Medicine, University of Alberta, Edmonton, AB Canada; 3 Department of Medicine, University of British Columbia, Arthritis Research Canada, Vancouver, BC Canada; 4 Department of Medicine, University of Ottawa, Ottawa, ON Canada

**Keywords:** Immune checkpoint inhibitors, IrAE, ICI, Needs assessment, Pediatrics, Rheumatic immune related adverse events

## Abstract

**Background:**

The use of immune checkpoint inhibitor (ICI) therapy is increasing in pediatric oncology. ICIs can cause rheumatic-immune related adverse events (Rh-irAEs) such as inflammatory arthritis and myositis. Few case reports detail Rh-irAEs and their management in the pediatric population. Our objective was to assess the familiarity of pediatric rheumatologists (PRs) worldwide with Rh-irAEs, gauge confidence in managing these conditions, and identify knowledge gaps to guide future educational efforts.

**Methods:**

We circulated an online survey to 2084 PRs via the “Dr. Peter Dent Pediatric Rheumatology Bulletin Board.” Responses were collected from June 2024 to September 2024. We collected data on practitioner demographics, knowledge of ICIs and Rh-irAEs, confidence in managing Rh-irAEs, and preferred educational resources.

**Results:**

Sixty-nine participants responded, of which 55 (80%) were PRs from academic centers. Despite global distribution, 56 (81%) responses came from North America. Thirty-four (49%) respondents were not aware of ICIs and their related mechanisms, indications, and side effects, and 40 (58%) were not familiar with irAEs. Fifty-five (80%) had never managed a patient with Rh-irAEs. Among those who had (14/69, 21%), the median number of cases managed was 2.0 (IQR 0.0). Thirty-nine respondents were “not confident at all” managing Rh-irAEs, 34 were “not confident at all” managing pre-existing autoimmune diseases (PAD) in ICI users, and 46 were “not confident at all” advising oncology colleagues on initiating or discontinuing ICIs in the context of Rh-irAEs or pre-existing autoimmune diseases (PAD). No respondents felt “completely confident” managing these conditions. Participants identified knowledge gaps in long-term management, acute management, and recognition and diagnosis. Forty-three indicated the need for pediatric-specific clinical guidelines. Of the 14 respondents with clinical experience treating Rh-irAEs, treatment varied, with 4 using nonsteroidal anti-inflammatory drugs, 3 using prednisone, and 4 combining prednisone with methotrexate. Long-term management also varied, with 5 using methotrexate, and 3 using tumor necrosis factor inhibitors.

**Conclusions:**

Significant knowledge gaps and a lack of confidence exist among PRs managing ICI-related Rh-irAEs. As ICI use increases in pediatric oncology, PRs’ exposure to Rh-irAEs will follow. Targeted educational programs and clinical guidelines may be valuable to address these gaps.

**Supplementary Information:**

The online version contains supplementary material available at 10.1186/s12969-025-01127-x.

## Background

Immune checkpoint inhibitors (ICIs) have emerged as a novel treatment for many cancers transforming patient outcomes in previously untreatable malignancies. Unlike conventional chemotherapy, which have the secondary effect of immunosuppression, ICIs increase anti-tumor activity by blocking intrinsic down-regulators of the immune system, thereby activating the immune system and enabling T cells to mount a robust anti-tumor response [1]. The use of ICIs is limited by autoimmune toxicities, called immune-related adverse events (irAEs), which occur due to an imbalance of unchecked T-cell activation and the resultant inflammation of non-tumor tissue [2, 3].

In adults exposed to ICIs, irAEs have been reported across a wide range of organ systems and include rheumatic irAEs (Rh-irAE) such as inflammatory arthritis, myositis, sarcoidosis, and vasculitis [4]. Treatment of adult Rh-irAEs is based on case reports, case series and physician gestalt, and has largely mimicked treatment of idiopathic diseases with similar phenotypes. With increasing clinical experience, optimal care pathways and consensus statements have been developed, and small clinical trials comparing different treatment strategies are underway [5].

The experience of ICI therapy in pediatrics has been limited. ICIs have been used in pediatric cancers since 2015 and are increasing. Approved pediatric indications include primary and adjuvant treatment of melanoma, Hodgkin’s lymphoma, primary mediastinal large B-cell lymphoma, Merkel cell carcinoma, and microsatellite instability-high or mismatch repair deficient colorectal cancer. Currently, ICIs are used in a minority of pediatric malignancy cases, making true incidence of their current use unknown. A retrospective cohort of 109 patients looked at the efficacy and safety of PD-1 inhibitors and described the distribution of tumor groups treated: Hodgkin lymphoma (28.0%), primary mediastinal large B cell lymphoma (2.2%), NK/T-cell lymphoma (11.8%), bone and soft tissue tumors (20.4%), central nervous system tumors (6.5%), and melanoma (2.2%) [6]. Although ICIs have been successful in treating adult solid tumors, their application in pediatrics has been less effective. This has prompted some to theorize that a relative lack of novel mutations in tumor cells may be the reason [7].

Currently, there is limited experience with pediatric irAEs, particularly Rh-irAE, which, in the pediatric literature is limited to case reports and a recent single-institution retrospective cohort study at a pediatric cancer center that evaluated 50 pediatric and young adult patients treated with off-label ICIs. Within this cohort, patients received treatment for a wide range of solid tumors (*n* = 28), neuro-oncologic tumors (*n* = 20), and hematologic malignancies (*n* = 2). In this cohort, 22/50 (44.0%) experienced irAEs, with GI toxicity being the most common overall [8, 9].

Similarly, there is little to guide optimal management of pediatric toxicities, and currently, treatment decisions are primarily based on experiences with adult patients, with suggested management algorithms based on the Rh-irAE severity, as categorized by the Common Terminology Criteria for Adverse Events (CTCAE) grading system [10, 11].

The extent of the pediatric rheumatology community’s experience in the developing field of Rh-irAEs is unknown. With the increasing use of ICIs in pediatrics, we anticipate a rise in Rh-irAEs, which will need to be recognized and managed by pediatricians and pediatric rheumatologists.

Our aim is to conduct a needs assessment to evaluate the familiarity of pediatric rheumatologists (PRs) worldwide with Rh-irAEs, gauge their confidence in managing these conditions, and identify key knowledge gaps for targeted educational intervention.

## Methods

We circulated a 21-question online survey distributed to 2084 PRs via the “Dr. Peter Dent Pediatric Rheumatology Bulletin Board”, an international listserv of pediatric rheumatologists that serves as a forum for discussion of difficult clinical cases and research endeavours. The survey questions (Appendix A) were adapted from a previous needs assessment conducted among adult rheumatologists in Canada in 2019 [11]. Responses were collected from June 2024 to September 2024. The study received approval from the Ethics Review Board of The University of British Columbia (Ethics Approval #H23-03354). Implied individual consent was obtained. There was no patient involvement.

We collected data on practitioner demographics, knowledge of ICIs and Rh-irAEs, previous experience in managing irAEs, the number of patients with irAEs seen, and the distribution of disease manifestations (e.g., inflammatory arthritis, vasculitis, connective tissue diseases). We also assessed practitioner confidence levels in managing irAEs and preferences for educational resources. Participants were unable to skip questions and were instructed not to complete the survey more than once.

Descriptive statistics were used to summarize the data. Categorical variables are presented as number (percentage), and continuous variables are presented as mean or median (interquartile range), as appropriate.

## Results

A total of 69 responses were received of potential 2084 PRs, for a response rate of 3.3%. Among these, 55/69 (79.7%) were from PRs from academic centers, while 9/69 (13.0%) were from community practices. The demographics of the respondents reflect a wide range of clinical experience (Table 1: Respondent Demographics, Knowledge & Confidence Assessment). Twenty-four (34.8%) reported having over 20 years of clinical experience, and 54/69 (78.3%) indicated that they spend more than 75% of their practice in pediatric rheumatology. Although the survey was circulated to an international cohort, 56/69 (81.1%) respondents were from North America, with 36/69 (52.2%) from the USA, 19/69 (27.5%) from Canada, and 1/69 (1.4%) from Mexico. Other countries included 4/69 (5.8%) from Türkiye, and 2/69 (2.9%) from Brazil. Spain, Thailand, Croatia, Germany, Palestine, and New Zealand all had one respondent (1.4%) each.

**Table 1:**
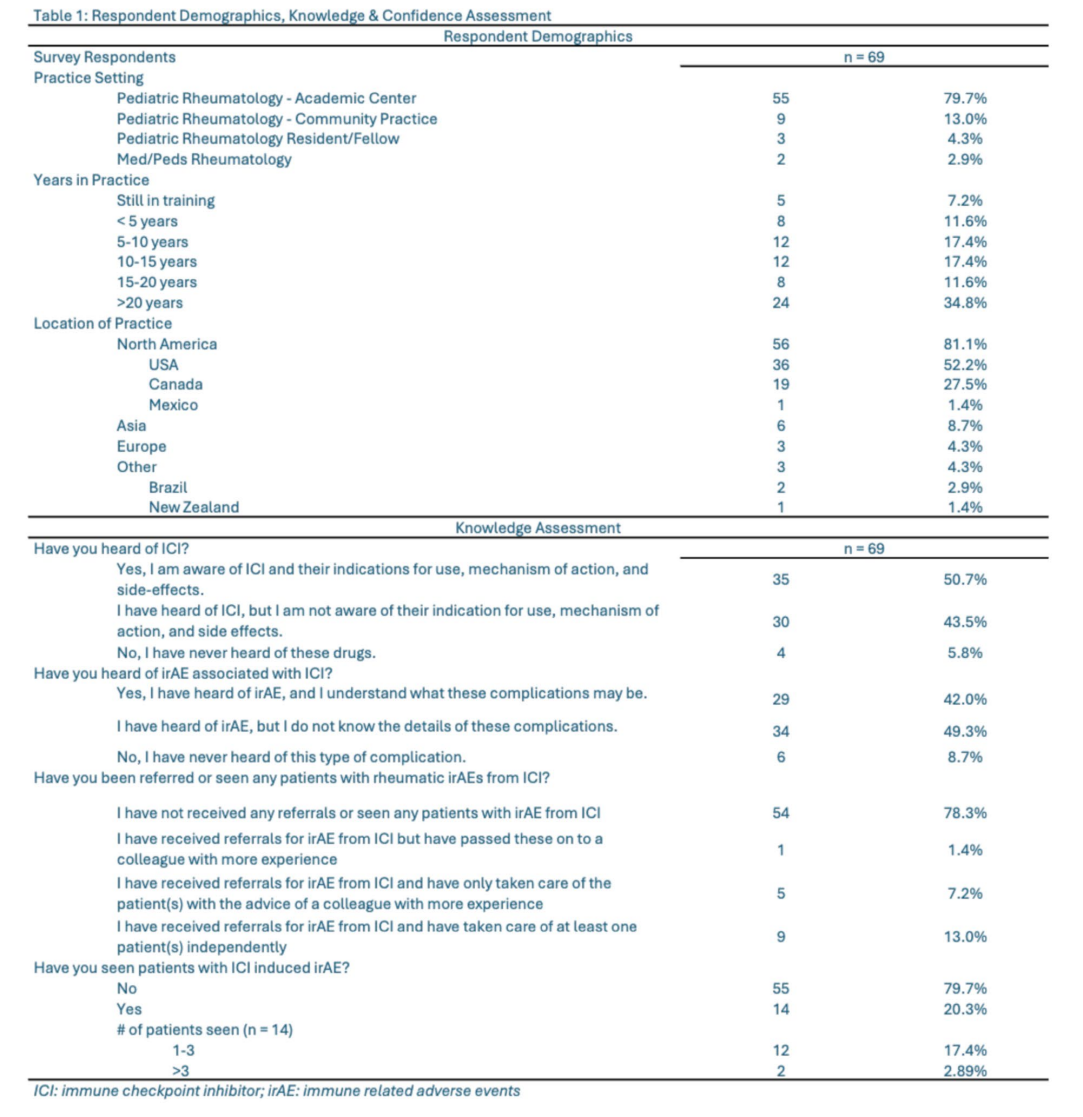
Respondent demographics, knowledge & confidence assessment

**Fig. 1:**
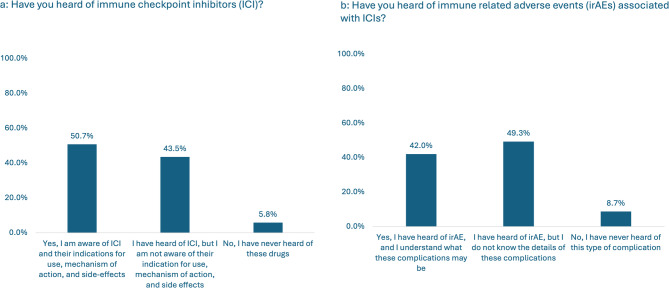
**a**, **b**: Immune Checkpoint Inhibitor Awareness

Despite most respondents regularly attending rheumatology conferences (53/69; 76.8% attended American College of Rheumatology Convergence), there was limited awareness of ICIs. Thirty-four (49.3%) of 69 respondents were not aware of ICIs and their related indications, mechanisms of action, and potential side effects (Fig. 1a: Immune Checkpoint Inhibitor Awareness) and 54/69 (78.3%) had never received referrals for patients with Rh-irAEs. Twenty-nine (42.0%) were aware of irAEs and understood their implications (Fig. 1b: Immune Checkpoint Inhibitor Awareness). Among those who had seen patients with Rh-irAEs, 12/14 (85.7%) reported seeing only 1–3 patients. One physician whose primary practice is in a combined adult/pediatric rheumatology practice reported having seen over 10 patients. The median number of patients seen was 2.0, with an interquartile range of 0.0.

**Fig. 2:**
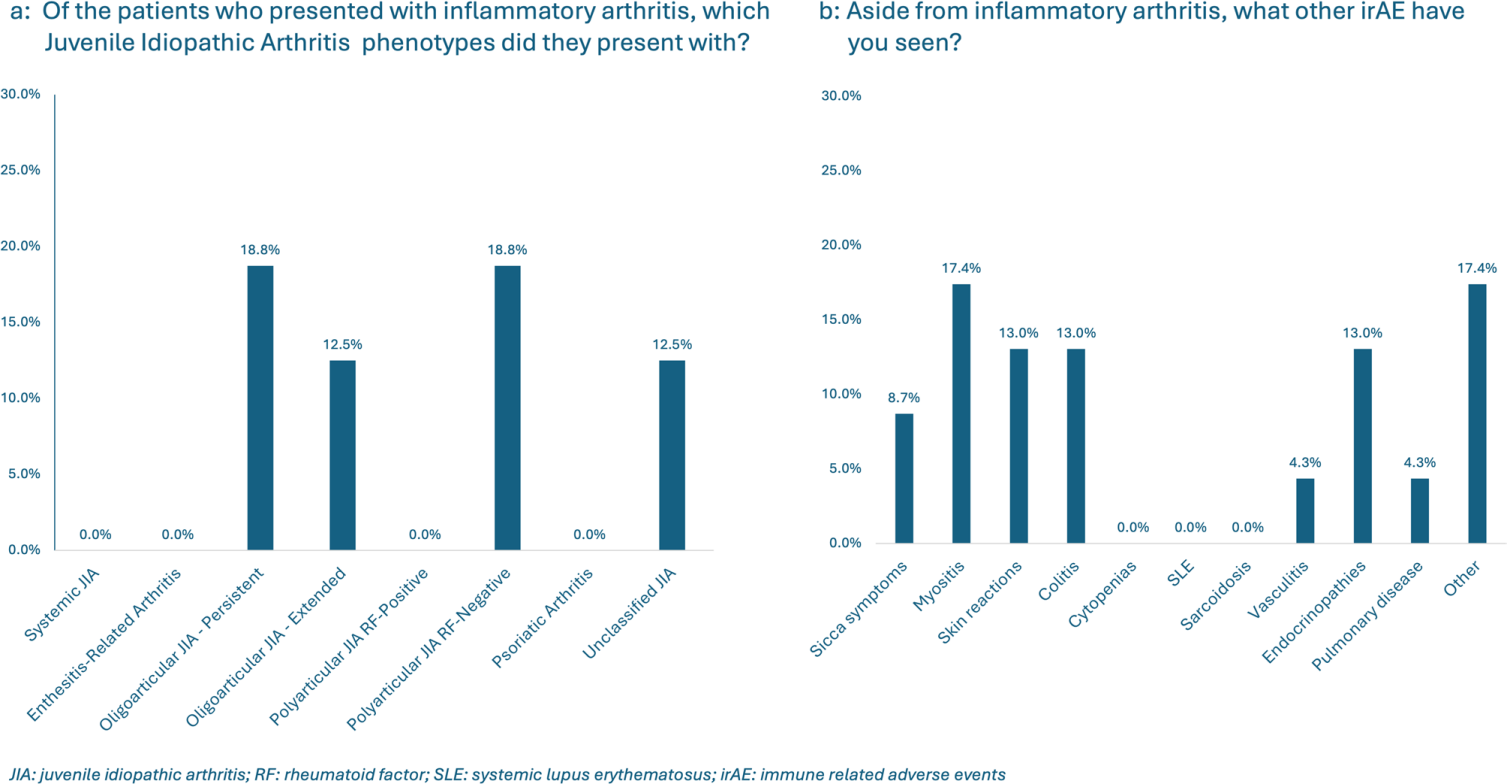
**a**,** b**: Manifestations of Rheumatic Immune Related Adverse Event

Of those who had experience with Rh-irAEs, 7/14 (50.0%) reported that more than 80% of the presentations seen were inflammatory arthritis, with polyarticular rheumatoid factor-negative and oligoarticular juvenile idiopathic arthritis being the most common subtypes, accounting for 18.8% each (Fig. 2a: Manifestations of Rheumatic Immune Related Adverse Events). Twenty-three non-articular irAEs were reported, with the most frequently observed non-articular irAEs being myositis (4/23, 17.4%), followed by colitis (3/23, 13.0%), skin reactions (3/23, 13.0%), endocrinopathies (3/23, 13.0%), sicca symptoms (2/23, 8.7%), vasculitis (1/23, 4.3%), pulmonary disease (1/23, 4.3%), and other non-specified symptoms (4/23, 17.4%) (Fig. 2b: Manifestations of Rheumatic Immune Related Adverse Events). 8/14 (57.1%) respondents reported that their patients had to stop or hold their ICI treatment due to irAEs.

**Fig. 3:**
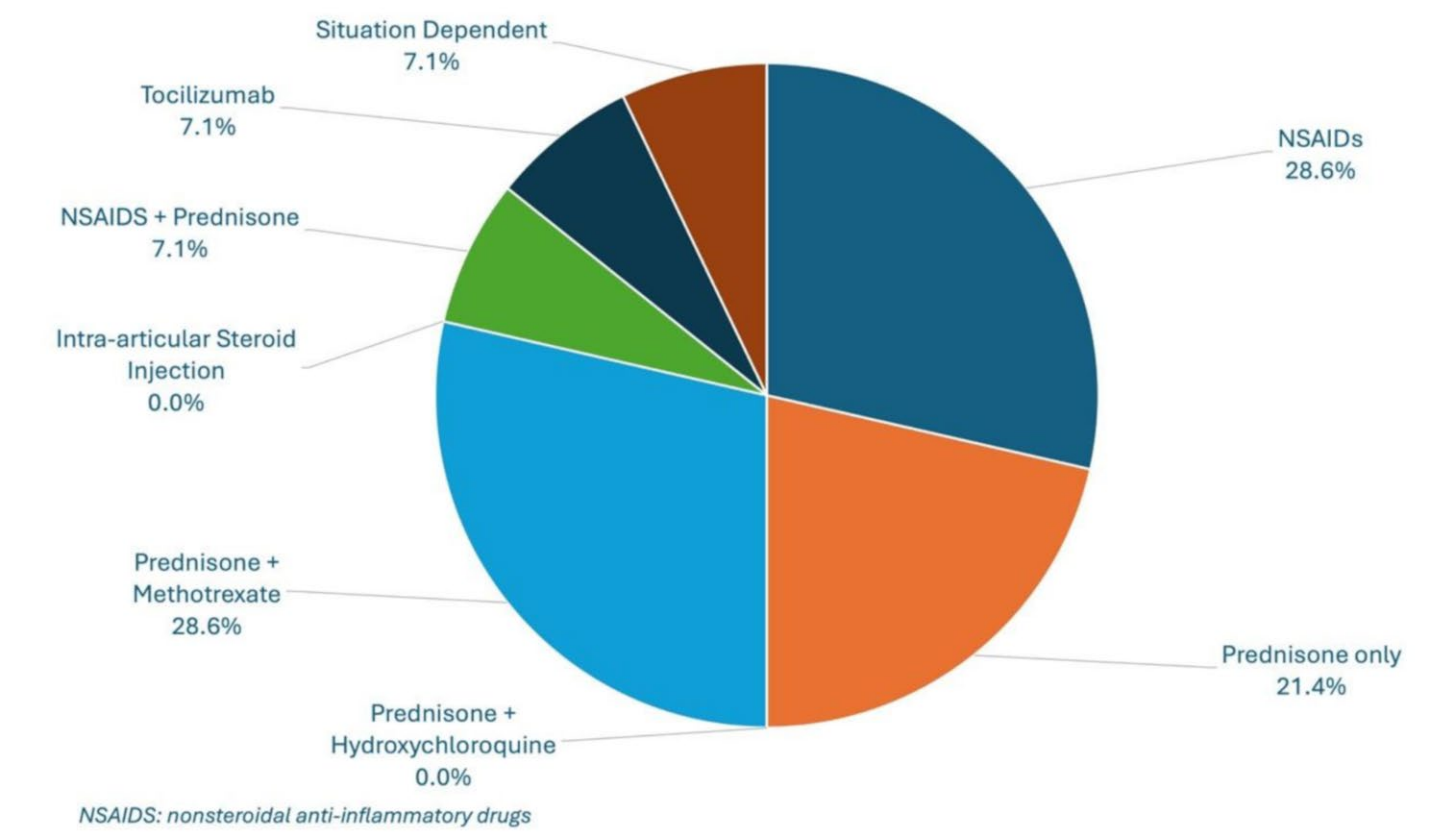
Initial Treatment Patterns

Of the 14 experienced respondents, initial treatment patterns revealed variability: 4/14 (28.6%) utilized nonsteroidal anti-inflammatory drug (NSAID) monotherapy, 3/14 (21.4%) used prednisone monotherapy, 4/14 (28.6%) combined prednisone with methotrexate, and 1/14 (7.1%) respondent each reported using anakinra, and prednisone in combination with NSAIDs. None of the respondents had used intra-articular corticosteroid injections or a combination of prednisone with hydroxychloroquine (Fig. 3: Initial Treatment Patterns).

Long-term management exhibited similar variability. Only 10/14 (71.4%) of experienced respondents had managed long-term ICI-associated inflammatory arthritis. Of these, 5/10 (50%) used methotrexate, 3/10 (30%) used a tumor necrosis factor (TNF)-inhibitor, 1/10 (10%) used tocilizumab, and 1/10 (10%) used prednisone monotherapy.

Seven (50.0%) of 14 experienced respondents had developed a referral relationship with their local oncologists, and 8/14 (57.1%) had been consulted on stopping, holding or resuming ICIs in the setting of irAE. Two (14.3%) had encountered resistance from their oncology colleagues for the use of methotrexate or other disease-modifying antirheumatic drugs in patients receiving ICIs. Three (21.4%) respondents were consulted on whether to start an ICI in patients with pre-existing autoimmune disease (PAD).

**Fig. 4:**
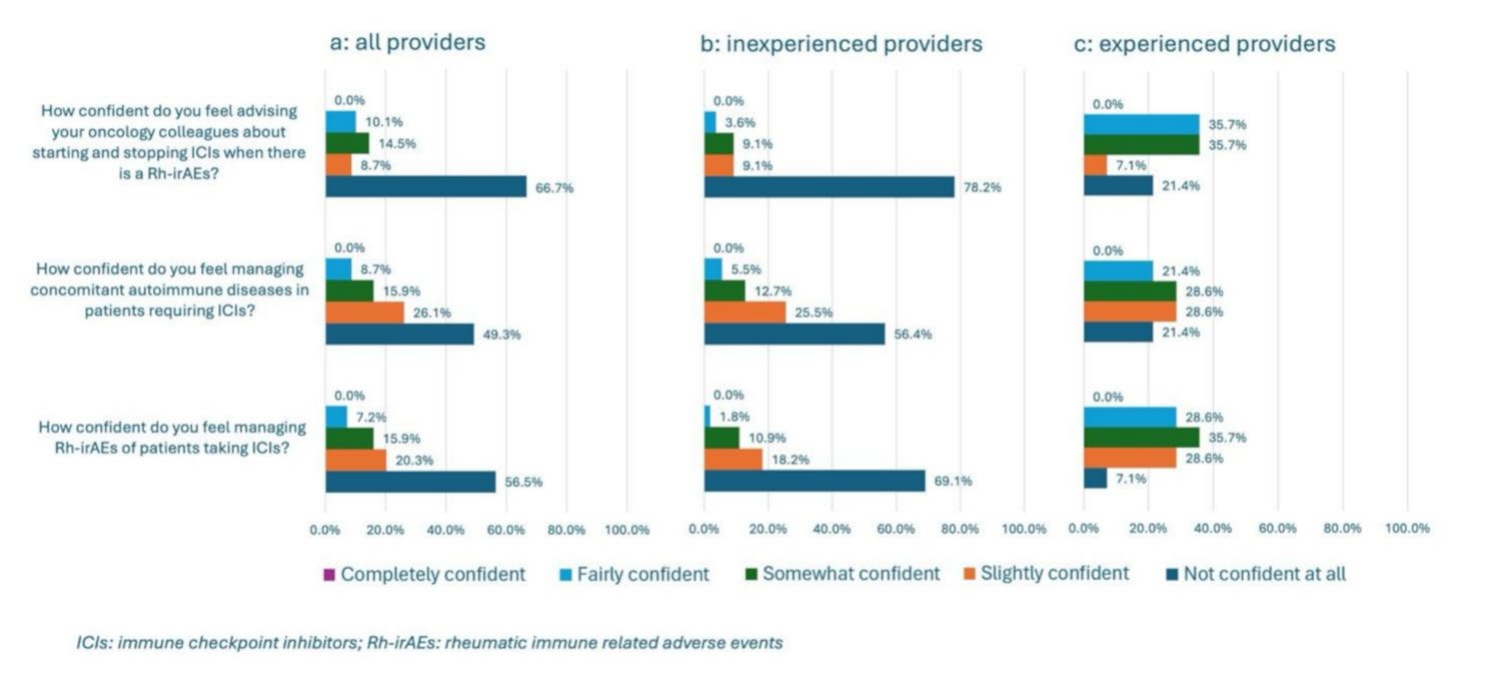
**a**,** b**,** c**: Confidence Assessment

Regarding confidence in management, overall, 39/69 (56.5%) respondents were “not confident at all” managing Rh-irAEs, 34/69 (49.3%) were “not confident at all” managing PAD in ICI users, and 46/69 (66.7%) were “not confident at all” advising oncology colleagues on initiating or discontinuing ICIs in the context of Rh-irAEs or PADs (Fig. 4a: Confidence Assessment).

Of respondents who had no experience with Rh-irAE management, 38/55 (69.1%) were “not confident at all” managing Rh-irAEs, 31/55 (56.4%) were “not confident at all” managing PAD in ICI users, and 44/55 (78.2%) were “not confident at all” advising oncology colleagues on initiating or discontinuing ICIs in the context of Rh-irAEs or PADs (Fig. 4b: Confidence Assessment).

Of respondents with prior experience managing Rh-irAEs, 1/14 (7.1%) was “not confident at all” managing Rh-irAEs, 3/14 (21.4%) were “not confident at all” managing PAD in ICI users, and 3/14 (21.4%) were “not confident at all” advising oncology colleagues on initiating or discontinuing ICIs in the context of Rh-irAEs or PADs (Fig. 4c: Confidence Assessment).

**Fig. 5:**
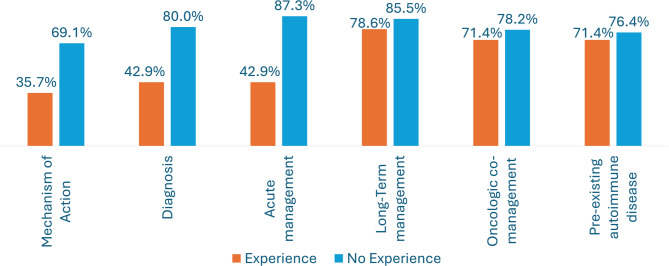
Knowledge Gaps

Knowledge gaps regarding Rh-irAEs were observed, with 59/69 (85.5%) respondents identifying gaps in long-term management, 55/69 (79.7%) in acute management, 51/69 (73.9%) in recognition and diagnosis of Rh-irAEs, 44/69 (63.8%) in mechanisms of action of ICIs, 53/69 (76.8%) in managing PAD, and 54/69 (78.3%) in the interplay of rheumatic and oncologic co-management (Fig. 5: Knowledge Gaps). Additionally, 43/69 (62.3%) respondents expressed a need for a pediatric-specific clinical practice guideline or consensus statement for the management of Rh-irAEs. When stratified by experience level, 6/14 (42.9%) respondents with experience and 37/55 (67.3%) with no experience expressed a need for pediatric-specific clinical practice guidelines.

**Fig. 6:**
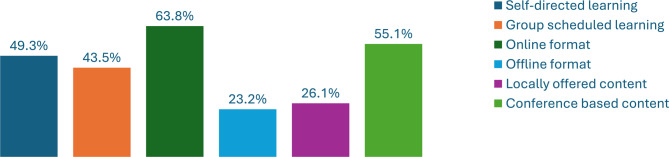
Educational Assessment

Sixty-three (91.3%) respondents expressed a willingness to participate in educational activities related to the recognition and management of Rh-irAEs, with 44/69 (63.8%) preferring online formats (e.g., learning modules, podcasts, webinars), while 34/69 (49.3%) showed interest in self-directed learning, and 30/69 (43.5%) desired group-scheduled activities (Fig. 6: Educational Assessment). There was a preference for conference-based content (55.1%) over locally offered content (26.1%). Six respondents indicated no interest in educational activities, five of whom had no previous clinical experience.

## Discussion

This study highlights significant gaps in awareness, experience, and confidence of PRs in managing Rh-irAEs. With the growing use of ICIs in pediatric oncology, there is a critical need to address these gaps through education, research, and knowledge dissemination, as nearly half (34/69, 49.3%) of all respondents were unfamiliar with ICIs and their associated adverse effects. Furthermore, PRs have had minimal to no exposure to Rh-irAEs, with most respondents having never seen a case. These findings emphasize the need for the development of educational interventions/tools and clinical practice guidelines and are in line with findings from a survey of adult rheumatologists at the advent of ICI indications for adult cancers [11].

In adults, Rh-irAEs can mimic idiopathic diseases that are not seen in children, such as polymyalgia rheumatica-like syndrome, remitting seronegative symmetrical synovitis with pitting edema, and gout [12–14]. Currently, it is unclear if these toxicities will also occur in the pediatric population. If they do, this will need to be a targeted area of education for pediatric practitioners in close collaboration with adult rheumatology as these conditions are not typically seen in pediatrics.

Confidence in managing Rh-irAEs was notably low among respondents, with more than half (39/69; 56.5%) indicating they were “not confident at all” in handling Rh-irAEs. This lack of confidence extended to advising oncology colleagues and presents a critical gap in interdisciplinary care. Strong collaborations between pediatric oncologists and rheumatologists are essential, with irAEs presenting a symbiotic learning opportunity between pediatric rheumatologists and oncologists.

The variability in treatment approaches observed among PRs reflects a lack of consensus amongst practitioners in management on irAEs. Although the European League Against Rheumatism published statements outlining points to consider in the diagnosis and management of ICI induced Rh-irAEs in adults, a majority of respondents indicated a need for pediatric-specific guidelines [5]. Such guidelines would help unify treatment approaches and allow further study into optimal treatment protocols.

Respondents expressed an interest in targeted educational programs in various formats to address the identified knowledge gaps. The widespread interest in conference-based educational content further emphasizes the need for professional organizations to consider comprehensive Rh-irAE-focused content in their programs.

The findings of this study parallel those observed in a prior Canadian survey of adult rheumatologists, which similarly identified knowledge gaps, low confidence levels, and variability in the management of Rh-irAEs [11]. However, the pediatric rheumatology community faces additional challenges, with an even greater proportion of respondents lacking familiarity with ICIs and their associated toxicities (49.3% in pediatrics vs. 30.9% in adults). Clinical experience with Rh-irAEs was also lower, with 54/69 (78.3%) of PRs having never received a referral for a patient with Rh-irAEs, compared to 89.7% of adult rheumatologists having seen fewer than five cases. The variability in treatment approaches observed among PRs underscores the absence of standardized management protocols. While some practitioners relied on NSAIDs or prednisone monotherapy, others incorporated methotrexate or biologics, with no clear consensus on the most effective strategy. Interestingly, no respondents reported using hydroxychloroquine for the management of Rh-irAEs, despite hydroxychloroquine being commonly used for Rh-irAEs in adult rheumatology [5, 15]. PRs expressed a strong desire for targeted educational initiatives, with 91.3% indicating interest in learning opportunities, compared to 59.0% in the adult study.

A key strength of this study is that it provides valuable insights into the current landscape of pediatric Rh-irAE management, highlighting significant gaps in awareness and confidence while also identifying opportunities for targeted educational interventions. This study also underscores the need for interdisciplinary collaboration between PRs and oncologists, most notably establishing referral pathways and shared decision-making frameworks; as well as necessary collaboration between adult and pediatric rheumatologists.

Although disseminated internationally, our study was limited by its low levels of participation outside of North America, thus representing predominantly North American perspectives and experiences. Our study also had a low response rate, and due to this may not accurately reflect the knowledge of PRs. Those who responded may have had a particular interest in ICIs and Rh-irAEs, leading to potential selection bias. Conversely, those who were less familiar with the topic may have been less likely to complete the survey, potentially underestimating knowledge gaps. Due to anonymity, emails from participants were not collected; thus, we were unable to verify that participants did not complete the survey more than once, however we feel that the risk of this is low.

## Conclusions

This study provides an important initial assessment of the pediatric rheumatology community’s familiarity with the management of Rh-irAEs. Our findings reveal significant knowledge gaps and variability in treatment approaches, underscoring the need for educational initiatives and the development of consensus guidelines tailored to pediatric rheumatology patients. As the use of ICIs continues to expand in pediatric oncology, efforts to bridge these gaps will be essential to ensure that PRs are well equipped to provide optimal care for patients with Rh-irAEs. Future research should focus on seeking perspectives from the broader international pediatric rheumatology community, building evidence-based treatment protocols, fostering collaboration between oncologists and pediatric rheumatologists and adult rheumatologists, and creating educational programs that enhance the recognition and management of these novel disease entities.

## Electronic supplementary material

Below is the link to the electronic supplementary material.

Supplementary Material 1

## Data Availability

No datasets were generated or analysed during the current study.

## Abbreviations

CTCAE: Common terminology criteria for adverse events
ICI: Immune checkpoint inhibitors
IQR: Interquartile range
irAE: Immune related adverse events
NSAID: Nonsteroidal anti-inflammatory drug
PRs: Pediatric rheumatologists
PAD: Pre-existing autoimmune disease
Rh-irAE: Rheumatic immune related adverse events
TNF: Tumor necrosis factor
