# Supplementary material for: Immune checkpoint inhibitors and the pediatric rheumatologist: a pediatric needs assessment
Source: Pediatr Rheumatol Online J. 2025 Jul 15;23:72. doi: 10.1186/s12969-025-01127-x (PMC12261645; doi:10.1186/s12969-025-01127-x)
Supplement: Supplementary file 1 — Supplementary Material 1 [file 12969_2025_1127_MOESM1_ESM.docx]

Appendix A: Survey Questions

1. What best describes your primary practice setting?
   1. Pediatric Rheumatology - Academic Center
   2. Pediatric Rheumatology - Community Practice
   3. Adult Rheumatology
   4. Pediatric Rheumatology Resident/Fellow
   5. Med/Peds Rheum Resident/Fellow
   6. Med/Peds Rheumatology - Academic Center
   7. Med/Peds Rheumatology - Community Practice
   8. Other (please specify)
2. How many years have you been in medical practice?
   1. Still in training
   2. < 5 years
   3. 5-10 years
   4. 10-15 years
   5. 15-20 years
   6. >20 years
3. What percentage of your practice is pediatric rheumatology?
   1. <25%
   2. 25-50%
   3. 50-75%
   4. >75%
4. Where is your country of practice? (Free text)
5. Which rheumatology conferences do you regularly attend? (choose all that apply)
   1. Canadian Rheumatology Association (CRA)
   2. American College of Rheumatology (ACR)
   3. European Alliance of Associations for Rheumatology (EULAR)
   4. Pediatric Rheumatology European Society (PReS)
   5. Pediatric Rheumatology Symposium (PRYSM)
   6. Asian Pacific League of Associations for Rheumatology (APLAR)
   7. African League Against Rheumatism (AFLAR)
   8. Pan-American League of Associations for Rheumatology (PANLAR)
   9. Childhood Arthritis and Rheumatology Research Alliance (CARRA)
   10. Other (please specify)
6. Have you heard of immune checkpoint inhibitors (ICI)?
   1. Yes, I am aware of ICI and their indications for use, mechanism of action, and side-effects
   2. I have heard of ICI, but I am not aware of their indication for use, mechanism of action, and side effects
   3. No, I have never heard of these drugs
7. Have you heard of immune related adverse events (irAE) associated with ICI?
   1. Yes, I have heard of irAE, and I understand what these complications may be
   2. I have heard of irAE, but I do not know the details of these complications
   3. No, I have never heard of this type of complication
8. Have you been referred or seen any patients with rheumatic immune related adverse events (irAEs) from ICI?
   1. I have not received any referrals or seen any patients with irAE from ICI
   2. I have received referrals for irAE from ICI but have passed these on to a colleague with more experience
   3. I have received referrals for irAE from ICI and have only taken care of the patient(s) with the advice of a colleague with more experience
   4. I have received referrals for irAEs from ICI and have taken care of at least one patient(s) independently
9. How many patients have you seen with immune checkpoint inhibitor (ICI) induced immune-related adverse events (irAE)?
   - 1. 0
     2. 1-3
     3. 4-6
     4. 7-9
     5. >10
   1. Of the patients you have seen, how many have presented with inflammatory arthritis?
      1. None
      2. <20%
      3. 20-50%
      4. 50-80%
      5. >80%
   2. Of the patients who presented with inflammatory arthritis, which JIA phenotypes did they present with? (choose all that apply)
      1. Systemic JIA Enthesitis Related Arthritis
      2. Oligoarticular JIA - Persistent
      3. Oligoarticular JIA - Extended
      4. Polyarticular JIA - RF Positive
      5. Polyarticular JIA - RF Negative
      6. Psoriatic Arthritis
      7. Unclassified JIA
      8. Not applicable
   3. Aside from inflammatory arthritis, what other irAE have you seen? (choose all that apply)
      1. Sicca symptoms
      2. Myositis
      3. Skin reactions
      4. Colitis
      5. Cytopenias
      6. SLE
      7. Sarcoidosis
      8. Vasculitis
      9. Endocrinopathies
      10. Pulmonary disease
      11. Other (please specify)
      12. No other irAEs seen
   4. What percentage of patients that you have seen have had to stop or hold their ICI because of a rheumatic immune related adverse event (irAE)?
      1. None
      2. < 20%
      3. 20-50%
      4. 50-80%
      5. >80%
   5. Have you or your group developed a referral relationship with your oncology colleagues for patients developing IRAEs?
      1. Yes
      2. No
   6. Have you been asked by your oncology colleagues to give advice about when to stop and/or resume ICI in the presence of IRAEs?
      1. Yes
      2. No
   7. Have you been asked by your oncology colleagues to give advice about whether to start ICI in a patient with well controlled (with or without medication) pre-existing autoimmune disease?
      1. Yes
      2. No
   8. Have you been asked by your oncology colleagues to give advice about whether to start ICI in a patient with active concomitant autoimmune disease?
      1. Yes
      2. No
   9. Have you found resistance from your oncology colleagues for the use of methotrexate and other DMARDs (excluding Prednisone) in patients receiving ICI?
      1. Yes
      2. No
10. What would be your general initial management of rheumatic irAEs with ICI?
    1. NSAIDs
    2. Prednisone only
    3. Prednisone + Hydroxychloroquine
    4. Prednisone + Methotrexate
    5. Intra-articular Steroid Injection
    6. NSAIDS + Prednisone
    7. Actemra
    8. Situation Dependent
11. What is your most commonly used immunosuppressant for long-term management of patients with rheumatic complications of ICI?
    1. Prednisone
    2. Hydroxychloroquine
    3. Methotrexate
    4. TNF inhibitors
    5. Tocilizumab
    6. I have never managed such patients
12. Are you aware of the Common Terminology Criteria for Adverse Events (CTCAE) grading system that can be used for immune-related adverse event?
    1. I am not familiar with the CTCAE gradings
    2. I am familiar with the CTCAE gradings, however have not used them in practice
    3. I am familiar with the CTCAE gradings and have used them in practice
13. Have you heard of CAN-Rio, a Canadian Research Group of Rheumatology in Immuno-Oncology?
    1. Yes
    2. No
14. Which of the following resources are you aware of? (choose all that apply)
    1. CanRIO Learning Modules
    2. CanRIO Case Rounds
    3. CanRIO Website
    4. EULAR Guidelines for the diagnosis and management of IRAEs
    5. None
    6. Other (Please specify)
15. How confident do you feel managing rheumatic IRAE of patients taking ICI?
    1. Not confident at all
    2. Slightly confident
    3. Somewhat confident
    4. Fairly confident
    5. Completely confident
16. How confident do you feel managing concomitant autoimmune diseases in patients requiring ICI?
    1. Not confident at all
    2. Slightly confident
    3. Somewhat confident
    4. Fairly confident
    5. Completely confident
17. How confident do you feel advising your oncology colleagues about starting and stopping ICI when there is a rheumatic IRAE?
    1. Not confident at all
    2. Slightly confident
    3. Somewhat confident
    4. Fairly confident
    5. Completely confident
18. Do you think there is a need for a pediatric specific clinical practice guideline or consensus statement for management of rheumatic irAEs associated with ICI?
    1. Yes
    2. No
    3. I don't know
19. What knowledge gaps do you have around immune related adverse events associated with immune checkpoint inhibitors? (choose all that apply)
    1. Mechanism of Action of ICIs
    2. Recognition and Diagnosis of irAEs
    3. Acute management of irAEs
    4. Long-Term management of irAEs
    5. Interplay of rheumatic and oncologic co-management
    6. Management of children with pre-existing autoimmune disease and ICI
    7. Other (please specify)
20. Would you be interested in participating in education activities pertaining to recognition and management of ICI-induced irAE?
    1. Very interested
    2. Moderately interested
    3. Somewhat interested
    4. Not interested
21. What format would you like to see for educational activity pertaining to recognition and management of ICI-induced irAE? (choose all that apply)
    1. Self-directed learning. (e.g. learning modules, podcasts, etc.)
    2. Group scheduled learning. (e.g. webinars, grand rounds, etc.)
    3. Online format. (e.g. learning modules, podcasts, webinars, etc.)
    4. Offline format. (e.g. conference workshops, grand rounds, etc.)
    5. Locally offered content. (e.g. departmental rounds, local workshops, etc.)
    6. Conference based content. (e.g. conference workshops, breakout rooms, etc.)
    7. Other (please specify)
    8. Society Guidelines
    9. Not interested
